# Quantitative Genetics of Microbiome Mediated Traits

**DOI:** 10.1101/2024.12.16.628599

**Published:** 2024-12-20

**Authors:** Bob Week, Peter L. Ralph, Hannah F. Tavalire, William A. Cresko, Brendan J. M. Bohannan

## Abstract

Multicellular organisms host a rich assemblage of associated microorganisms, collectively known as their “microbiomes”. Microbiomes have the capacity to influence their hosts’ fitnesses, but the conditions under which such influences contribute to evolution are not clear. This is due in part to a lack of a comprehensive theoretical framework for describing the combined effects of host and associated microbes on phenotypic variation. Here we begin to address this gap by extending the foundations of quantitative genetic theory to include host-associated microbes, as well as alleles of hosts, as factors that explain quantitative host trait variation. We introduce a way to partition host-associated microbiomes into componenents relevant for predicting a microbiome-mediated response to selection. We then apply our general framework to a simulation model of microbiome inheritance to illustrate principles for predicting host trait dynamics, and to generalize classical narrow and broad sense heritabilities to account for microbial effects. We demonstrate that microbiome-mediated responses to host selection can arise from various transmission modes, not solely vertical, with the contribution of non-vertical modes depending on host life history. Our work lays a foundation for integrating microbiome-mediated host variation and adaptation into our understanding of natural variation.

## Full Text Availability

The license terms selected by the author(s) for this preprint version do not permit archiving in PMC. The full text is available from the preprint server.

